# Supplementary material for: Dissecting the molecular organization of the translocon-associated protein complex
Source: Nat Commun. 2017 Feb 20;8:14516. doi: 10.1038/ncomms14516 (PMC5321747; doi:10.1038/ncomms14516)
Supplement: Supplementary Information — Supplementary figures, supplementary table and supplementary references. [file ncomms14516-s1.pdf]

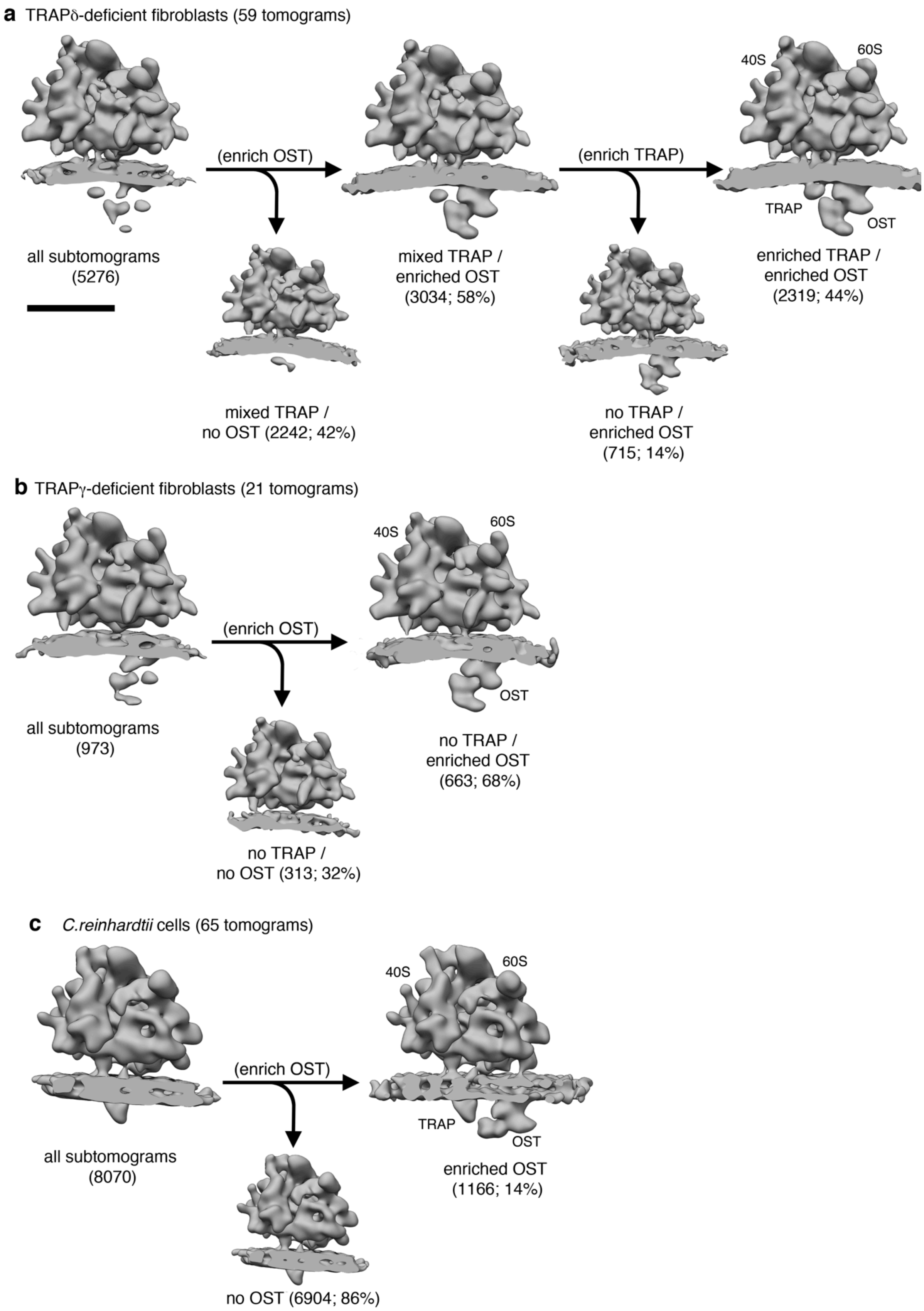
a TRAPδ-deficient fibroblasts (59 tomograms)
(enrich OST)
(enrich TRAP)
40S
60S
TRAP
OST
all subtomograms (5276)
mixed TRAP / enriched OST (3034; 58%)
enriched TRAP / enriched OST (2319; 44%)
mixed TRAP / no OST (2242; 42%)
no TRAP / enriched OST (715; 14%)
b TRAPγ-deficient fibroblasts (21 tomograms)
(enrich OST)
40S
60S
OST
all subtomograms (973)
no TRAP / enriched OST (663; 68%)
no TRAP / no OST (313; 32%)
c C.reinhardtii cells (65 tomograms)
(enrich OST)
40S
60S
TRAP
OST
all subtomograms (8070)
enriched OST (1166; 14%)
no OST (6904; 86%)

Supplementary Figure 1. Computational sorting of translocon populations. a) For the TRAPδ-deficient fibroblasts, two consecutive classification steps were used to enrich subtomograms depicting ribosomes bound to the OST- (left) and TRAP-containing (right) translocon. The density of ribosomes bound to the OST-lacking translocon complex (lower row, left) suggests a similar TRAP abundance in this population as for OST-containing translocon complexes. Scale bar: 20 nm. **b)** For the TRAPγ-deficient fibroblasts, subtomograms depicting ribosomes bound to the OST-containing translocon were enriched. Further classification did not yield classes with defined density for TRAP. **c)** For the *C. reinhardtii* cells, subtomograms depicting ribosomes bound to the OST-containing translocon were enriched. All densities were filtered to 30 Å resolution.

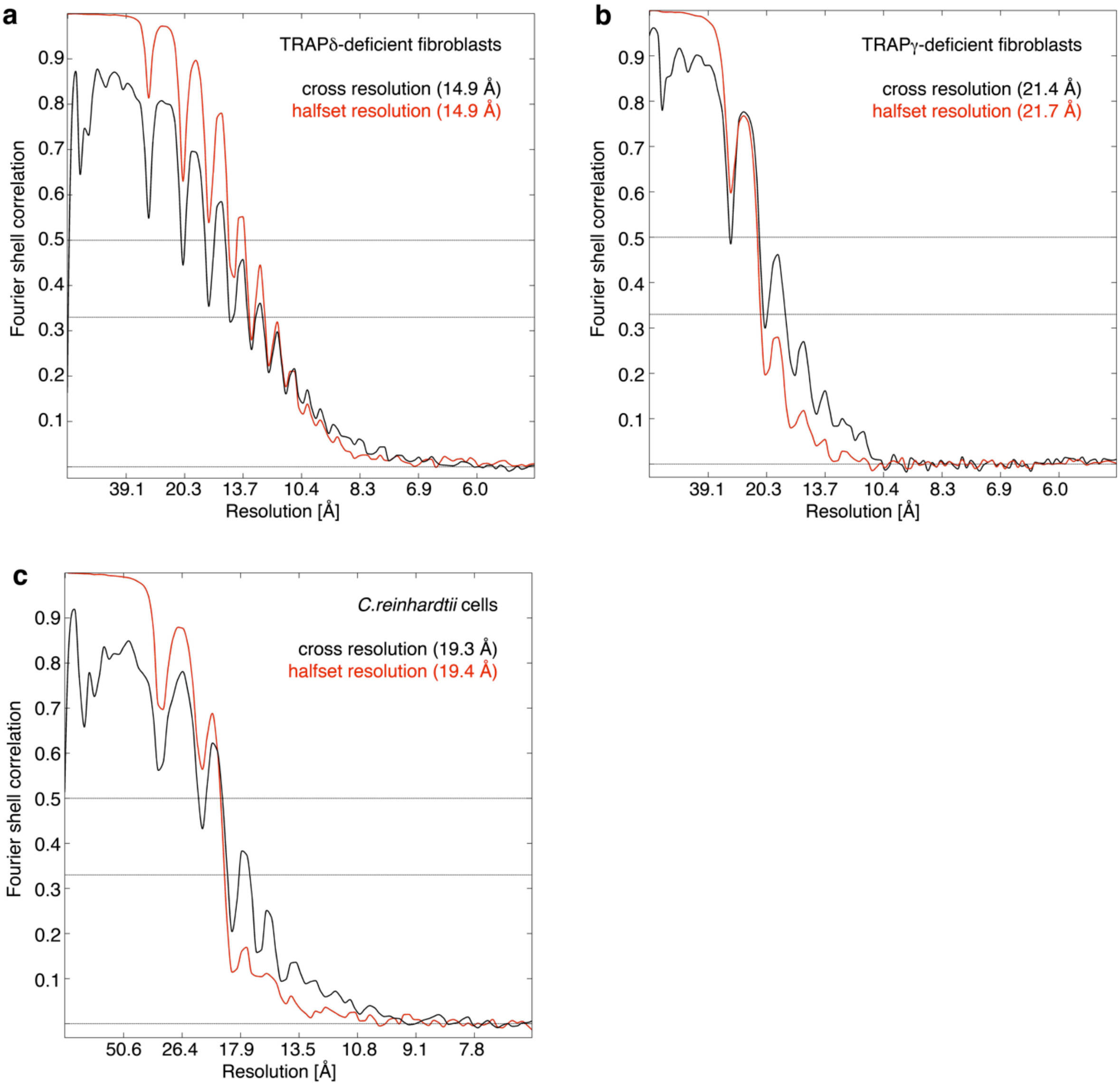

Supplementary Figure 2. Resolution estimation via Fourier shell correlation. Fourier shell correlation curves for the final subtomogram averages obtained for the TRAPδ- (**a**) and TRAPγ-deficient patient primary fibroblasts (**b**) and *C. reinhardtii* cells (**c**). The resolutions of the final subtomogram averages were estimated by Fourier shell correlation of two halves of the data (red, 0.5 FSC criterion) and Fourier shell cross resolution (black, 0.33 FSC criterion) against a single particle reconstruction of the human (EMD 5592; **a,b**) or wheat germ (EMD 1780; **c**) 80S ribosome.

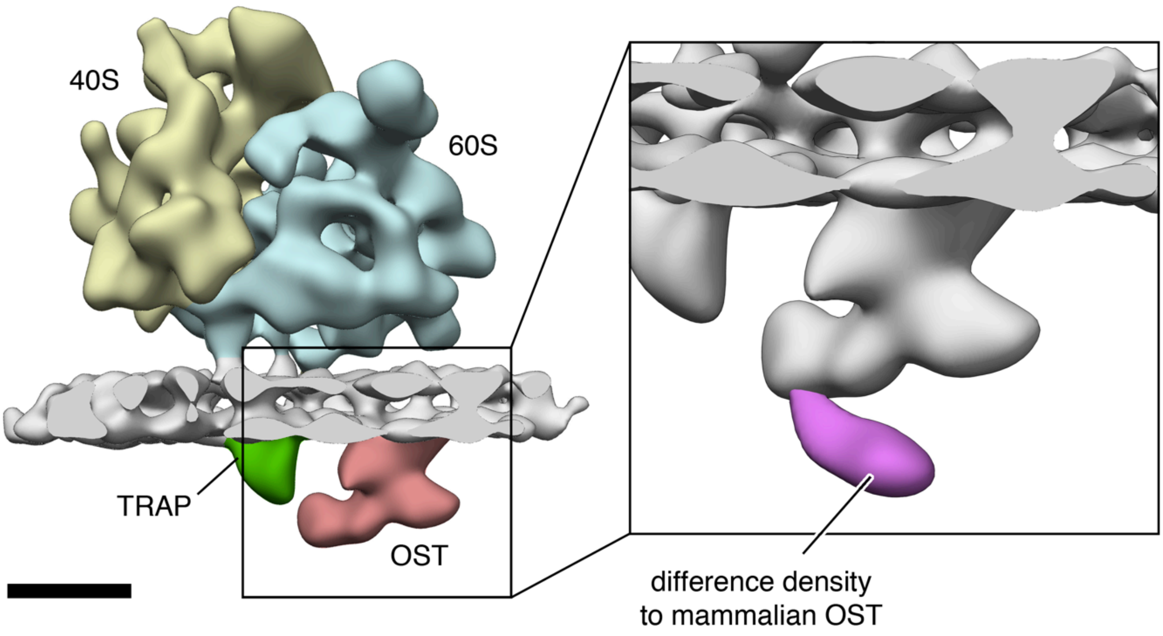

Supplementary Figure 3. Algal OST lacks a lumenal lobe compared to mammalian OST. Left: Subtomogram average of the *C. reinhardtii* ribosome (large subunit: blue, small subunit: yellow) bound to the OST-containing translocon, with the membrane bilayer (grey), TRAP (green) and OST (red) resolved. 1166 subtomograms were averaged. Scale bar: 10 nm. Right: Zoomed view of the indicated area with the difference density map (magenta) between *C. reinhardtii* and mammalian OST complexes superimposed on the subtomogram average (grey).

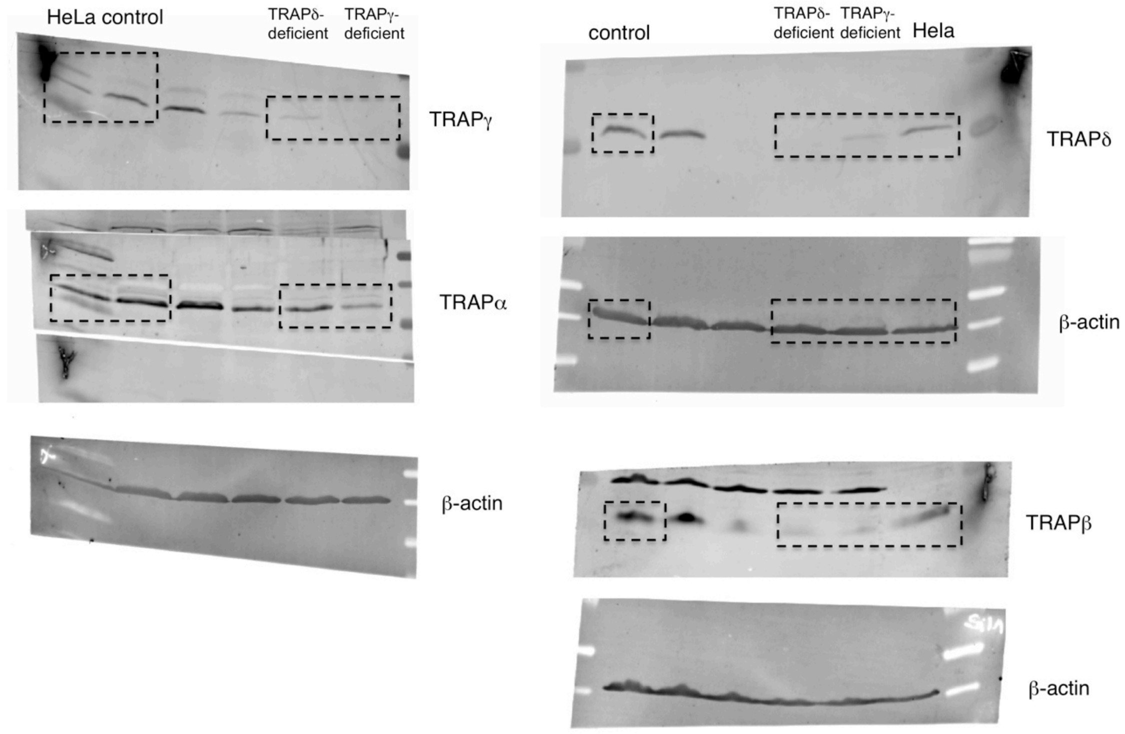

Supplementary Figure 4. Full Western blot scans from Figure 2 and Figure 3. Cropped areas are indicated.

Supplementary Table 1. List of antibodies used in this study.

Primary antibody	Western blot of	Dilution	Company	Catalog number	Citation
TRAPα	HeLa cells	1:500			Pfeffer, S. *et al.*, *Nat Commun* **5**, (2014)[1]
TRAPβ	HeLa cells	1:500			Pfeffer, S. *et al.*, *Nat Commun* **5**, (2014)[1]
TRAPγ	HeLa cells	1:500	Sigma	HPA014906	Pfeffer, S. *et al.*, *Nat Commun* **5**, (2014)[1]
TRAPδ	HeLa cells	1:500	Sigma	HPA045209	Pfeffer, S. *et al.*, *Nat Commun* **5**, (2014)[1]
Sec61α	HeLa cells	1:200			Lang, S. *et al.*, *J. Cell. Sci*. **125**, (2012)[2]
Sec62	HeLa cells	1:500			Lang, S. *et al.*, *J. Cell. Sci*. **125**, (2012)[2]
TRAM	HeLa cells	1:500			Guth, S. *et al.*, *Eur. J. Biochem*. **271**, (2004) [3]
OST48	HeLa cells	1:250	Santa Cruz	sc-74408	Pfeffer, S. *et al.*, *Nat Commun* **5**, (2014)[1]
Ribophorin I	HeLa cells	1:500			Pfeffer, S. *et al.*, *Nat Commun* **5**, (2014)[1]
Sil1	Canine pancreatic rough microsomes	1:500			Weitzmann, A. *et al.*, *FEBS J*. **274**, (2007)[4]
ERj3	HeLa cells	1:500			Schorr, S. *et al.*, *J. Biol. Chem*. **290**, (2015)[3]
β-actin	HeLa cells	1:10.000	Sigma	A5441	Pfeffer, S. *et al.*, *Nat Commun* **5**, (2014)[1]
anti-rabbit IgG-Cy5	HeLa cells	1:1000	GE	PA45012	Pfeffer, S. *et al.*, *Nat Commun* **5**, (2014)[1]
anti-mouse IgG-Cy3	HeLa cells	1:1000	GE	PA43010	Pfeffer, S. *et al.*, *Nat Commun* **5**, (2014)[1]

Supplementary References

1 Pfeffer, S. *et al*. Structure of the mammalian oligosaccharyl-transferase complex in the native ER protein translocon. *Nat Commun* **5**, 3072 (2014).

2 Lang, S. *et al*. Different effects of Sec61alpha, Sec62 and Sec63 depletion on transport of polypeptides into the endoplasmic reticulum of mammalian cells. *J. Cell Sci.* **125**, 1958-1969 (2012).

3 Guth, S. *et al.* Protein transport into canine pancreatic microsomes: a quantitative approach. *Eur. J. Biochem.* **271**, 3200-3207 (2004).

4 Weitzmann, A., Baldes, C., Dudek, J. & Zimmermann, R. The heat shock protein 70 molecular chaperone network in the pancreatic endoplasmic reticulum - a quantitative approach. *The FEBS journal* **274**, 5175-5187 (2007).
